# Acute stressors do not impair short-term memory or attention in an aged mouse model of amyloidosis

**DOI:** 10.3389/fnbeh.2023.1151833

**Published:** 2023-05-12

**Authors:** Giuliana M. DiMarco, Breanna N. Harris, Alena V. Savonenko, Paul L. Soto

**Affiliations:** ^1^Department of Biological Sciences, Texas Tech University, Lubbock, TX, United States; ^2^Center for Molecular and Behavioral Neuroscience, Rutgers, The State University of New Jersey, Newark, NJ, United States; ^3^Department of Pathology, The Johns Hopkins University School of Medicine, Baltimore, MD, United States; ^4^Department of Psychology, Louisiana State University, Baton Rouge, LA, United States

**Keywords:** amyloidosis, predator odor, forced swim, aging, serial reaction time task, delayed match-to-position task, amyloid-beta, Alzheimer’s disease

## Abstract

Memory impairment in Alzheimer’s disease patients is thought to be associated with the accumulation of amyloid-beta peptides and tau proteins. However, inconsistent reports of cognitive deficits in pre-clinical studies have raised questions about the link between amyloid-beta and cognitive decline. One possible explanation may be that studies reporting memory deficits often involve behavioral assessments that entail a high stress component. In contrast, in tasks without a high stress component transgenic mice do not consistently show declines in memory. The glucocorticoid cascade hypothesis of aging and the vicious cycle of stress framework suggest that stress exacerbates dementia progression by initiating a cycle of hypothalamic-pituitary-adrenal axis activation and subsequent brain deterioration. Using the APPswe/PS1dE9 mouse model of amyloidosis, we assessed whether stressor exposure prior to testing differentially impaired cognitive performance of aged male and female mice. As part of a larger study, mice performed a delayed match-to-position (DMTP) or a 3-choice serial-reaction time (3CSRT) task. Unexpectedly, these mice did not exhibit cognitive declines during aging. Therefore, at 73 and 74 weeks of age, we exposed mice to a predator odor or forced swim stressor prior to testing to determine if stress revealed cognitive deficits. We predicted stressor exposure would decrease performance accuracy more robustly in transgenic vs. non-transgenic mice. Acute stressor exposure increased accuracy in the DMTP task, but not in the 3CSRT task. Our data suggest that acute stressor exposure prior to testing does not impair cognitive performance in APPswe/PS1dE9 mice.

## Introduction

Alzheimer’s disease (AD) is a progressive neurodegenerative disease affecting approximately six million people in the United States, with a financial burden amounting to $321 billion (Alzheimer’s Association, 2022). AD has been characterized by accumulation of amyloid-beta (Aβ) neuritic plaques and tau fibrillary tangles, cognitive impairment, and dysregulation of the hypothalamic-pituitary-adrenal (HPA) axis (Jack et al., 2018; Justice, 2018). Dysregulation of the HPA axis leads to increased levels of circulating glucocorticoids which, according to the glucocorticoid cascade hypothesis and the vicious cycle of stress framework, lead to changes in memory-related brain structures and further dementia progression (Sapolsky et al., 1986; Justice, 2018).

A variety of mouse models of AD have been developed to understand the role of Aβ plaques and tau fibrillary tangles in neuronal and cognitive dysfunction. One of the most frequently used AD mouse models is the APPswe/PS1dE9 (APP/PS1) mouse, a mouse model of amyloidosis (Jankowsky et al., 2001), which has been used in a variety of behavioral paradigms such as the Morris Water Maze (MWM), Novel Object Recognition (NOR) task, and operant tasks (Arendash et al., 2001; Savonenko et al., 2003, 2005; Jankowsky et al., 2004; Cao et al., 2007; Cheng et al., 2014; Webster et al., 2014; Harris et al., 2023; Soto et al., 2023). Despite the widespread use of APP/PS1 mice, memory impairment is not reported consistently across behavioral paradigms (see Webster et al., 2014).

One possible explanation for conflicting results in cognitive impairment in APP/PS1 mice could be differences in the behavioral paradigms in which these mice are tested (Butler-Struben et al., 2022). Deficits in APP/PS1 mice are often reported from behavioral assessments, such as the MWM and fear conditions tasks (auditory conditioning or shocks; Webster et al., 2014), both of which entail a high stress component (Arendash et al., 2001; Savonenko et al., 2005; Cao et al., 2007; Zhang et al., 2011). In contrast, in behavioral assessments without a high stress component, such as the NOR task, these mice do not consistently show declines in memory (Cheng et al., 2014; Sierksma et al., 2014; Harris et al., 2023). Stressor exposure, as well as glucocorticoid concentrations, can alter multiple aspects of physiology and behavior, including cognition (Roozendaal, 2002; Harris, 2020). Given the documented role of both stress and glucocorticoids on cognitive function and Aβ pathophysiology (e.g., glucocorticoid cascade hypothesis and the vicious cycle of stress; Sapolsky et al., 1986; O’Brien, 1997; Justice, 2018), and that we have previously found transgenic (Tg) APP/PS1 mice have higher post-stressor corticosterone than non-Tg mice (Harris et al., 2023), it is reasonable to question whether behavioral paradigm stressfulness plays a role in cognitive outcomes. The current study was part of a larger study to evaluate cognitive deficits in APP/PS1 mice in which Tg mice showed extensive brain amyloid beta concentration, but did not exhibit cognitive deficits through ∼71 weeks of age (Soto et al., 2023). Here, we evaluated whether stressor exposure immediately prior to operant task testing altered short-term memory and attention of aged male and female Tg and non-Tg APP/PS1 mice. Mice were trained on a delayed match-to-position (DMTP) or 3-choice-serial-reaction time (3CSRT) operant task using food reinforcement (e.g., Woolley and Ballard, 2005; Higgins and Breysse, 2008), tasks which test short-term memory and attention, respectively, and putatively involve less stress than water escape tasks. At 73 and 74 weeks of age, mice were exposed to predator odor or a swim stressor prior to one of the tasks (order of presentation counterbalanced across mice; see Supplementary Figure 1). The swim stressor mimics the physiological challenges experienced during MWM testing, while the predator odor exposure relies on an innate fear of predators (e.g., predator exposure increases activity of the HPA axis and increases vigilance behaviors; Harris and Carr, 2016). We predicted that stressor exposure would decrease performance accuracy in both tasks, and that deficits would be more pronounced in Tg compared to their non-Tg mice.

## Materials and methods

### Animals

B63C3-Tg (APPswe/PS1dE9) 85Dbo/Mmjax mice that express Mo/HuAPP695swe (a chimeric mouse/human amyloid precursor protein) and PS1-dE9 (mutant human presenilin 1) were purchased from The Jackson Laboratory (Bar Harbor, Maine; MMRRC Stock #34829). APPswe and PS1-dE9 are genetic mutations associated with AD and allow these mice to develop Aβ neuritic plaques between 6 and 7 months of age (Jankowsky et al., 2001, 2004; Reiserer et al., 2007). Mice were bred in-house (Soto et al., 2023) and genotyped around 4 weeks of age (Transnetyx, Inc., Cordova, TN, USA). Mice were singly housed in cages (Techniplast polysulfone, Blue Line 1285L, 35.56 cm L × 20.32 cm W, 13.97 cm H) lined with corn cob bedding and housing room lights remained on from 0700 to 1,900 every day.

### Stressor events

#### Predator odor exposure

A clean cage was lined with corn cob bedding. A 100% pure cotton ball soaked with 1 ml of Wolf Urine^1^ Store was placed in a 59 ml cup (Diamond Daily, BPA-free, Leak Resistant, Disposable Mini Cup, Jarden Home Brands, Fishers, IN, USA) on the right side of the cage (cup was covered with parafilm poked with holes). Mice were placed in the cage containing the wolf urine for 10 min. Immediately following exposure, mice were taken for behavioral testing. We have previously shown that exposure to predator odor increases glucocorticoids in APP/PS1 mice (Harris et al., 2023).

#### Swim test

A clear cylinder (Tritan, BPA-free, Beverage Dispenser, Buddeez, Inc., Wentzville, MO, USA), 6.62 L capacity, inner diameter 22.9 cm, was filled 5 cm from the top with clean water between 24 and 25°C. Mice were placed in the water for exactly 6 min. Mice were monitored throughout testing and were removed from the water immediately if the mouse’s head went under water for more than 3 s, if the mouse’s head went under water three times, or if the mouse’s entire body went under water (this occurred once). Following the forced swim task, mice were allowed to move freely within a warming cage heated by a 72-Watt light placed 12.6 cm from the cage floor for 5 min (temperature ranged from 29 to 58°C within different regions of the cage). Mice were then immediately placed in their respective chambers for operant testing. We have previously shown that swimming elevates glucocorticoids in mice (Harris et al., 2012).

#### Corticosterone measurement

Prior to euthanasia, an undisturbed blood sample was collected from the retro-orbital sinus for determination of baseline corticosterone. A few days later, mice were then exposed to either swim stressor or a predator odor stressor and a blood sample was collected immediately after. Both baseline and post-stressor plasma samples were analyzed for corticosterone concentration as we have done previously (Harris et al., 2023). Both stressors significantly elevated plasma corticosterone in these animals (Supplementary Figure 2). Data were analyzed by RM ANOVA with condition (baseline or post-stressor) as the repeated factor and genotype (and sex) as the fixed factor.

### Operant tasks

Food was restricted to 85% of total free-feeding weight and mice were fed standard rodent chow after operant testing each day. Water was available *ad libitum* in the home cage. Mice were tested 5 days per week between 0900 and 1,300 on one task, either the DMTP or 3CSRT task, from ∼16 to 72 weeks of age. At 73 and 74 weeks of age, mice were tested for the impact of stressor exposure on task performance as follows: the first 1–2 days of testing each week were conducted without any stressor exposure, the subsequent session (i.e., the 2nd or 3rd day of the week), stressor exposure (predator odor or forced swim; order counterbalanced across mice) preceded testing, and the remainder of sessions each week were conducted without any prior stressor exposure (Supplementary Figure 1A). Operant chambers in the same room were labeled for either male or female mice to avoid scent contamination. Each mouse was exposed to each stressor. Experimenters were not responsible for any subjective measurements about behavior as all data were automatically recorded by MedPC software. Note, behavioral data collected prior to stressor testing are presented elsewhere (Soto et al., 2023).

#### Delayed match to position (DMTP) task

Short-term memory was measured using a delayed match to position (DMTP) task. Operant chambers (ENV-307W, Med Associates, St. Albans, VT, USA) contained two retractable levers and a food cup between the two levers, into which 20-mg food pellets could be delivered via a food dispenser. Each session was limited to 60 trials or 35 min, whichever occurred first. Mice were trained to retrieve 20-mg food pellets readily from the food dispenser by delivering them at random. Next, mice were trained to lever press using food delivery as a reinforcer. Trials began with illumination of the house light followed by extension of one lever. If the mouse pressed the lever, it retracted, and a delay of 0.1, 1, 2, 4, 8, 16, or 24 s began. Following the delay period, a nose-poke entry triggered the extension of the two levers. If the mouse pressed the correct lever, a 20-mg food pellet was dispensed (Supplementary Figure 1B).

#### 3-choice serial-reaction time (3CSRT) task

Attention was measured using a 3-choice serial-reaction time (3CSRT) task (Higgins and Breysse, 2008; Tsutsui-Kimura et al., 2009). Operant chambers (ENV-307W, Med Associates, St. Albans, VT) contained 3 nose-poke-holes with LED lights on one wall and a pellet dispenser on the opposite wall. Each session was limited to 100 trials or 35 min, whichever occurred first. Initially 20-mg pellets were delivered into the food cup at random intervals until pellets were readily retrieved. Mice were trained in four stages before moving to the standard procedure. Stimulus duration decreased with each stage (stage 1: 10-s, stage 2: 5-s, stage 3: 3-s, stage 4: 2-s, standard procedure: 1-s). Stage advancement required greater than 80% accuracy and less than 30% omissions. Trials began with illumination of the house light and a 5-s pre-stimulus period. One of the three nose-poke holes was then illuminated, and a nose-poke response during the illumination or within 5-s of illumination produced delivery of a food pellet (Supplementary Figure 1C).

### Data analysis

#### DMTP

For the forced swim stressor regression analysis, data from 11 non-Tg female, 11 non-Tg male, 11 Tg female, and 11 Tg male mice were included. For the predator odor stressor regression analysis, data from 10 non-Tg female, 13 non-Tg male, 10 Tg female, and 10 Tg male mice were included.

For analysis of the delayed match-to-position (DMTP) data, a mixed effects logistic regression was conducted using the glmer command in the lme4 library (Bates et al., 2015) of the free open-source statistical language *R* (R Core Team, 2018) similar to that described previously (Wileyto et al., 2004; Bailey et al., 2018; Young, 2018). Because aggregating binomial outcomes risks misidentification of interactions (Dixon, 2008) and fails to retain information regarding differential sample size (due to attrition) as well as differential variability (due to individual differences or experimental control), we analyzed the choice data in binomial form. The repeated measures binomial outcome data collected in the DMTP and 3CSRT (see below) procedures necessitated the use of mixed effects logistic regression. It is important to note that a logistic regression analyses the relation between the log odds of an outcome (i.e., the log-transformed ratio of the probability of an outcome occurring to the probability of the outcome not occurring, which is unbounded, unlike percent correct and thus not subject to the same artifacts) and one or more predictors. Thus, the coefficients returned by the analyses conducted indicate the change in log odds of a correct response for each unit change in the continuous predictors or for the different levels of the categorical predictors. For ease of interpretation, results are visually presented in terms of proportion correct. For analysis of the DMTP data, three models were generated using every completed trial with the outcome being the trial outcome (correct = 1, incorrect = 0). In the first model (DMTP Model 1), the predictors used were Genotype (categorical, effect-coded; transgenic = 1 and non-transgenic = –1), session timing (categorical, dummy-coded; Before, On, or After the day of stressor exposure with Before coded as the reference level), and delay (continuous, log-transformed, and grand-mean centered; 0.1–24 s). In the second model (DMTP Model 2), the predictors were Genotype, session timing, delay, and stressor type (categorical, dummy-coded; Water vs. Odor with Water coded as the reference level). Finally, a third model (DMTP Model 3) was conducted using predictors of Genotype, session timing, delay, and Sex (categorical, dummy-coded; Female vs. Male with Female coded as the reference level). All models entailed a random coefficient of the delay variable by mouse. Models were subsequently compared using a chi-square test using the anova function of the car package (Fox and Weisberg, 2019) and model AIC values.

#### 3CSRT

For the swim stressor regression analysis, data from 12 non-Tg female, 11 non-Tg male, 9 Tg female, and 9 Tg male mice were included. For the predator odor stressor regression analysis, data from 10 non-Tg female, 10 non-Tg male, 9 Tg female, and 10 Tg male mice were included.

For analysis of the 3-choice-serial-reaction time (3CSRT) data, mixed effects logistic regression was used as described above. In the first model (3CSRT Model 1), the predictors used were Genotype and session timing. In the second model (3CSRT Model 2), the predictors were Genotype, session timing, and stressor type. Finally, a third model (3CSRT Model 3) was conducted using predictors of Genotype, session timing, and Sex. All models entailed a random intercept by mouse. As described above, models were subsequently compared using a chi-square test and model AIC values.

## Results

### DMTP

**FIGURE 1:**
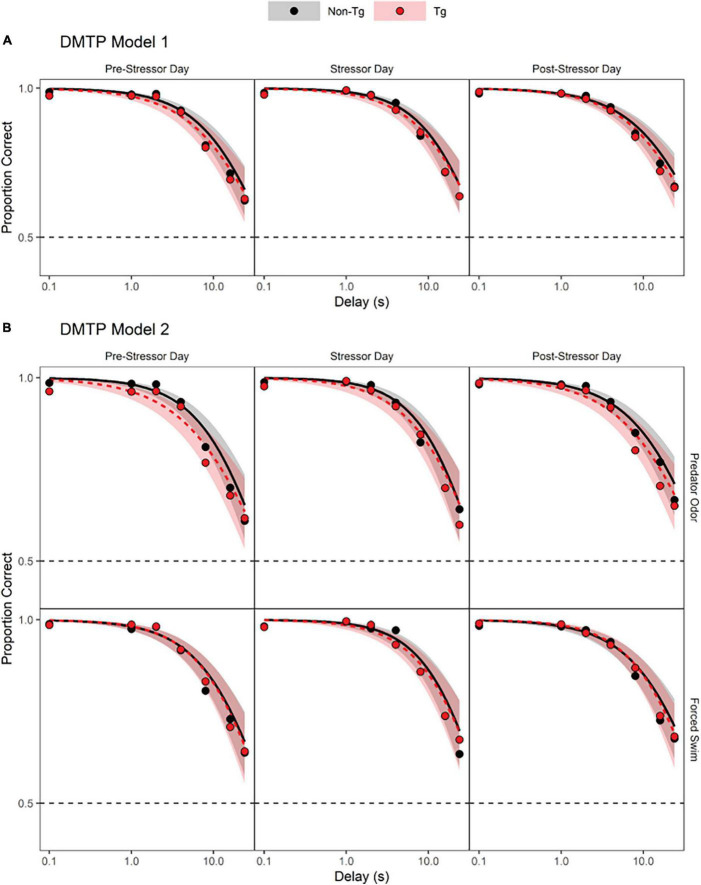
Delayed match-to-position (DMTP) results. **(A)** Predicted log odds of a correct response in the DMTP task based on a model that incorporated three predictors: delay (0.1–24 s; continuous, log-transformed, and centered), timing of session (before, on, or after the day of stressor exposure; dummy-coded with “on” as reference level) and genotype [transgenic (Tg) vs. non-transgenic (non-Tg); effect-coded: non-Tg = 1 and Tg = –1]. Each panel depicts the predicted log odds of a correct response as a function of delay (log scale) between sample lever presentation and the choice opportunity. Solid black lines indicate the model prediction for non-transgenic mice and gray shaded areas indicate the 95% confidence limits around the model prediction. Dashed red lines indicate the model prediction for transgenic mice and light red shaded areas in the 95% confidence limits around the model prediction. **(B)** Predicted log odds of a correct response in the DMTP task as a function of delay between sample presentation and choice opportunity based on a model that incorporated four predictors: delay (0.1–24 s; continuous, log-transformed, and centered), timing of session (before, on, or after the day of stressor exposure; dummy-coded with on as reference level) genotype [transgenic (Tg) vs. non-transgenic (non-Tg); effect-coded: non-Tg = 1 and Tg = –1], and stressor type (water vs. odor; dummy-coded with Before as reference level).

Results from the DMTP Model 1 analysis indicated that accuracy decreased significantly as the delay increased for both genotypes (Figure 1A and Table 1, DMTP Model 1, model term 3; Supplementary Figure 3 individual data). The intercept of the log odds of a correct response vs. delay relation was increased on stressor exposure days, relative to the intercept obtained on sessions prior to the stressor exposure day (Table 1, DMTP Model 1, model term 4), indicating that stressor exposure increased accuracy at short delays on the day of stressor exposure. In contrast, the intercept of the log odds correct vs. delay relation was not statistically significantly different on days after stressor exposure compared to the days before stressor exposure (Table 1, DMTP Model 1, model term 5). The effect of genotype was not statistically significant (Table 1, DMTP Model 1, model term 2), nor were any of the interaction terms involving genotype (Table 1, model terms 6–8, 11, and 12).

**TABLE 1:** Delayed match-to-position (DMTP) task results coefficient estimates (estimate), standard errors (SE) of the estimates, and resulting z-statistic (z) and associated *p*-values (p) from the multilevel logistic regression analysis of DMTP performance before, on and after stressor exposure days for each predictor variable (predictor).

Predictor	Estimate	SE	*z*	*p*
**DMTP Model 1 (AIC = 17,850)**				
(Intercept)	2.803	0.168	16.69	<0.001
Genotype (Tg)	-0.281	0.243	-1.15	0.249
Delay	-2.389	0.164	-14.59	<0.001
Session timing (on)	0.287	0.129	2.22	0.027
Session timing (after)	0.071	0.099	0.72	0.470
Genotype (Tg):delay	0.245	0.237	1.03	0.302
Genotype (Tg):session timing (on)	0.010	0.182	0.06	0.955
Genotype (Tg):session timing (after)	0.173	0.142	1.22	0.221
Delay:session timing (on)	-0.247	0.192	-1.29	0.198
Delay:session timing (after)	0.181	0.148	1.22	0.222
Genotype (Tg):delay:session timing (on)	0.056	0.272	0.21	0.838
Genotype (Tg):delay:session timing (after)	-0.252	0.213	-1.18	0.238
**DMTP Model 2 (AIC = 17844.8)**				
(Intercept)	2.735	0.174	15.76	<0.001
Genotype (Tg)	0.022	0.254	0.09	0.930
Delay	-2.340	0.225	-10.39	<0.001
Stressor type (odor)	0.245	0.212	1.16	0.248
Session timing (on)	0.627	0.243	2.59	0.010
Session timing (after)	0.312	0.213	1.46	0.144
Genotype (Tg):delay	-0.039	0.332	-0.12	0.907
Genotype (Tg):stressor type (odor)	-0.568	0.307	-1.85	0.064
Delay:stressor type (odor)	-0.341	0.290	-1.18	0.239
Genotype (Tg):session timing (on)	-0.201	0.343	-0.59	0.558
Genotype (Tg):session timing (after)	0.009	0.306	0.03	0.977
Delay:session timing (on)	-0.559	0.344	-1.63	0.104
Delay:session timing (after)	-0.071	0.309	-0.23	0.819
Stressor type (odor):session timing (on)	-0.607	0.340	-1.79	0.074
Stressor type (odor):session timing (after)	-0.265	0.280	-0.95	0.344
Genotype (Tg):delay:stressor type (odor)	0.613	0.421	1.46	0.145
Genotype (Tg):delay:session timing (on)	0.296	0.490	0.60	0.546
Genotype (Tg):delay:session timing (after)	-0.085	0.445	-0.19	0.848
Genotype (Tg):stressor type (odor):session timing (on)	0.490	0.482	1.02	0.309
Genotype (Tg):stressor type (odor):session timing (after)	0.211	0.398	0.53	0.597
Delay:stressor type (odor):session timing (on)	0.636	0.488	1.30	0.192
Delay:stressor type (odor):session timing (after)	0.427	0.370	1.16	0.248
Genotype (Tg):delay:stressor type (odor):session timing (on)	-0.593	0.696	-0.85	0.394
Genotype (Tg):delay:stressor type (odor):session timing (after)	-0.278	0.525	-0.53	0.596

Omission trials were excluded from analysis. For the simpler model (DMTP Model 1), the fixed effects portion of the model included estimates for the predictors of genotype (effect-coded: non-transgenic = −1, transgenic = 1), delay (0.1, 1, 2, 4, 8, and 16 24; continuous, centered, and log-transformed), session timing (before, on, or after the day of stressor exposure), and all possible interactions of the three predictors. For the more complex model (DMTP Model 2), the fixed effects portion of the model included estimates for the predictors of genotype (effect-coded: non-transgenic = −1, transgenic = 1), delay (0.1, 1, 2, 4, 8, and 16 24; continuous, centered, and log-transformed), session timing (before, on, or after the day of stressor exposure), and stressor type (odor or water), and all possible interactions of the four predictors. The random effects portion of both models included estimates of the coefficient of delay for each mouse. According to a comparison of AIC values and a model comparison via chi-square, the more complex model provided a better description of the results [χ^2^(12) = 29.106, *p* = 0.004].

Results from the DMTP Model 2 analysis indicated again that accuracy declined significantly as delay increased for both genotypes (Figure 1B and Table 1, DMTP Model 2, model term 3; Supplementary Figure 3 individual data). Similar to the results of the first model, the only other predictor that produced a statistically significant impact on accuracy was session timing with increased log odds of a correct response at short delays occurring on the days of stressor exposure, relative to sessions that occurred prior to the day of stressor exposure (Table 1, DMTP Model 2, model term 5). Although adding the type of stressor exposure to the model improved the model [lower AIC value for DMTP Model 2 than DMTP Model 1 and χ^2^(12) = 29.106, *p* = 0.004], the effect of stressor exposure was not statistically significant (Table 1, DMTP Model 2, model term 4), nor were any interactions involving stressor type significant (Table 1, DMTP Model 2, model terms 8, 9, 14–16, 19–24). Finally, the effect of genotype alone or in combination with other predictors did not meet criteria for statistical significance (Table 1, DMTP Model 2, model terms 2, 7, 8, 10, 11, 16–19, 23, and 24).

We also analyzed the potential impact of sex of the mice on DMTP performance by adding Sex as a predictor variable (DMTP Model 3), but this analysis did not reveal any significant effect of Sex alone or in combination with other predictors (Supplementary Table 1, model terms 6, 12–15, 18–24).

### 3CSRT

**FIGURE 2:**
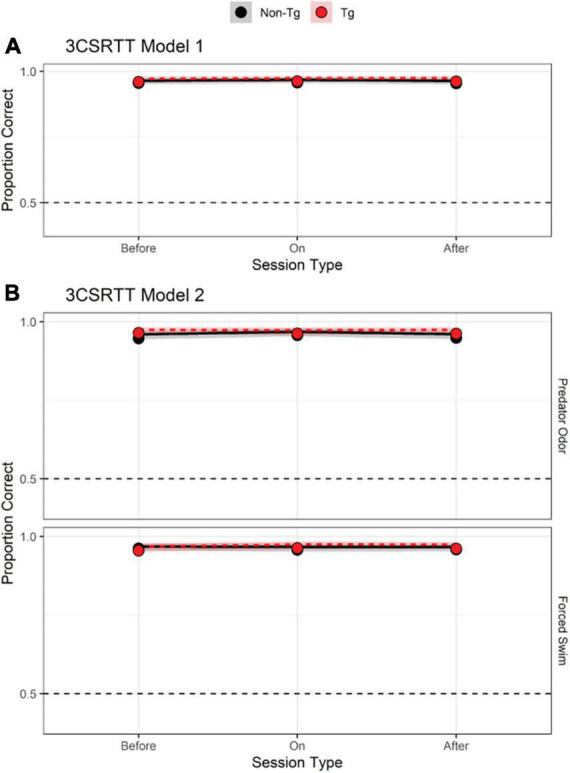
3-choice serial-reaction time (3CSRT) results. **(A,B)** Predicted log odds of a correct response in the 3CSRT task based on a model that incorporated two predictors: timing of session relative to stressor exposure (pre-stress, stressor day, or post-stressor exposure; dummy-coded with “on” as reference level) and genotype [transgenic (Tg) vs. non-transgenic (non-Tg); effect-coded: non-Tg = 1 and Tg = –1]. Other details as in Figure 1.

Results from the 3-choice-serial-reaction time (3CSRT) Model 1 analysis indicated no effect of genotype or session timing on accuracy (Figure 2A and Table 2, Model 1, model terms 2–6; Supplementary Figure 4 individual data) in the 3CSRT task. Adding stressor type as a predictor failed to produce a better model based on a comparison of AIC values (Figure 2B and Table 2) or chi-square test [χ^2^(12) = 7.011, *p* = 0.320] indicating that the simpler model provides a more parsimonious account of the results. Including Sex as a predictor did not reveal an effect of Sex on accuracy in the 3CSRT task alone or in combination with other predictors (Supplementary Table 2, model terms 5, 8–12).

**TABLE 2:** 3-choice serial-reaction time (3CSRT) task results.

Predictor	Estimate	SE	*z*	*p*
**3CSRT Model 1 (AIC = 8477.1)**				
(Intercept)	3.300	0.167	19.80	<0.001
Genotype (Tg)	0.217	0.258	0.84	0.399
Session timing (pre-stress)	0.112	0.118	0.95	0.344
Session timing (post-stress)	-0.012	0.096	-0.12	0.903
Genotype (Tg):session timing (on)	-0.016	0.180	-0.09	0.928
Genotype (Tg):session timing (after)	0.123	0.146	0.84	0.400
**3CSRT Model 2 (AIC = 8482.1)**				
(Intercept)	3.422	0.181	18.92	<0.001
Genotype (Tg)	-0.021	0.276	-0.07	0.941
Stressor type (odor)	-0.245	0.137	-1.79	0.074
Session timing (on)	-0.040	0.170	-0.24	0.814
Session timing (after)	-0.057	0.139	-0.41	0.684
Genotype (Tg):stressor type (odor)	0.477	0.208	2.29	0.022
Genotype (Tg):session timing (on)	0.294	0.265	1.11	0.266
Genotype (Tg):session timing (after)	0.295	0.206	1.43	0.153
Stressor type (odor):session timing (on)	0.291	0.237	1.23	0.218
Stressor type (odor):session timing (after)	0.073	0.190	0.38	0.702
Genotype (Tg):stressor type (odor):session timing (on)	-0.599	0.362	-1.66	0.098
Genotype (Tg):stressor type (odor):session timing (after)	-0.329	0.288	-1.14	0.253

Coefficient estimates (estimate), standard errors (SE) of the estimates, and resulting z-statistic (z) and associated *p*-values (p) from the multilevel logistic regression analysis of 3CSRT performance before, on, and after stressor exposure days for each predictor variable (predictor). Omission trials were excluded from analysis. For the simpler model (3CSRT Model 1), the fixed effects portion of the model included estimates for the predictors of genotype (effect-coded: non-transgenic = −1, transgenic = 1), session timing (before, on, or after the day of stressor exposure), and the possible interactions. For the more complex model (3CSRT Model 2), the fixed effects portion of the model included estimates for the predictors of genotype, session timing, and stressor type (odor or water), and all possible interactions of the three predictors. The random effects portion of both models included separate intercepts for each mouse. According to a comparison of AIC values and a model comparison via chi-square, the increased number of predictors was not justified by a significant improvement in description [χ^2^(12) = 7.011, *p* = 0.320].

## Discussion

Memory impairment in mouse models of amyloidosis has been studied using a variety of behavioral paradigms; however, inconsistent deficits in pre-clinical studies and conflicting results from clinical studies on the amyloidogenic pathway have led to questions about the role of Aβ in AD-associated cognitive deficits (Webster et al., 2014; Makin, 2018). We hypothesized that the stress inherent in water maze tasks, in which deficits have consistently been reported (e.g., Savonenko et al., 2005; Zhang et al., 2011), might play a role in revealing deficits in aged APP/PS1 mice which had not previously shown deficits in working memory or attention operant tasks (Soto et al., 2023). Therefore, the current study evaluated whether acute stressor exposure prior to testing altered short-term memory and attention of aged male and female non-Tg and Tg APP/PS1 mice. We predicted that pre-task stressor exposure would decrease performance accuracy in these otherwise putatively low-stress operant tasks, and that these deficits would be more pronounced in Tg compared to non-Tg littermate mice. Contrary to our predictions, we found that stressor exposure did not reduce accuracy in either the DMTP or 3CSRT tasks. On stressor days, both Tg and non-Tg mice showed equivalent small *increases* in accuracy in the DMTP task and no change in the 3CSRT task, although accuracy was so high in the 3CSRT task, improvement likely could not be detected. Acute stressor exposure prior to testing does not impair short-term memory or attention in this mouse model of amyloidosis as tested here. Additionally, we did not see sex differences in outcomes, despite female Tg mice having higher Aβ than male TG mice (Soto et al., 2023). However, the role of sex differences in AD mouse model data is often inconsistent (e.g., Fisher et al., 2018). Our data also suggest that the high-stress nature of some behavioral tasks alone cannot account for the differences in memory impairment seen across behavioral tasks using APP/PS1 mice, although it may be that stress during the task, such as what occurs in the Morris Water Maze (MWM) task, is critical for deficits to appear.

Memory performance can be impacted differently depending on the timing and duration of stressor events and resulting hormone concentrations (Sandi and Pinelo-Nava, 2007; Klier and Buratto, 2020); therefore, it is important to consider how experimental design may have impacted the results of this study. We exposed mice to a forced swim test to mimic the elevated blood glucocorticoid levels (i.e., stress) induced by MWM sessions. It is likely that blood glucocorticoids are elevated during at least part of MWM sessions, in which mice are exposed to up to 6 min of swimming per day punctuated by handling. Blood glucocorticoid levels are detectable after ∼3–5 min and remain elevated for ∼30–60 min following both predator odor exposure and forced swim exposure (Harris et al., 2012). Across humans and rodents, post-stressor levels of glucocorticoids are related to decreases in cognitive performance (Sauro et al., 2003). This relationship has also been reported in the MWM, as Harrison et al. (2009) found that corticosterone levels 30-min after the final MWM test session were positively correlated with escape latency, swim path length, and search errors. We previously found that aged APP/PS1 mice have a greater corticosterone response to predator odor than non-Tg mice (Harris et al., 2023). Therefore, it may be that the corticosterone response to forced swim in APP/PS1 mice (see Supplementary Figure 2 for corticosterone response to swimming) may also be enhanced and relate to decreased spatial memory performance. However, we found that acute stressor exposure prior to testing slightly improved rather than impaired accuracy in the short-term memory task (results which align with previous studies we performed in humans during aging; Harris et al., 2022). Thus, our data do not fit the vicious cycle of stress framework, but do align with the inverted U hypothesis (Sandi and Pinelo-Nava, 2007; Northoff and Tumati, 2019). It may be that the stressors and glucocorticoid responses here fall on the ascending limb of the inverted U-shaped curve.

Another possibly important difference between MWM and operant task assessments employed in the current study is that in MWM, animals experience a limited number of training sessions whereas in the current study, the animals experienced many experimental sessions. Tasks such as the MWM do not require extensive repeated training periods (typically only 5–6 days), whereas operant tasks often do (Woolley and Ballard, 2005; Vorhees and Williams, 2006; Higgins and Breysse, 2008) and it may be that extensive training ameliorates deficit development. However, Lonnemann et al. (2023) showed that brief (8 days) exposure every 3 months to the MWM alleviated some of the memory deficits seen in aged APP/PS1 mice, suggesting that minimal task exposure can reduce deficits. Consistent with this suggestion, APP/PS1 mice who began operant task training in a pairwise discrimination task at 3 months old, and likely had no Aβ accumulation, performed similarly to control mice, however, those who began training at 12 months of age, when Aβ accumulation is pronounced, exhibited impaired performance compared to control mice (Van den Broeck et al., 2021). Additionally, APP/PS1 mice trained on the operant task at 6 months of age, when moderate Aβ accumulation is expected, performed similarly to controls initially, but exhibited decreased performance in the reversal learning portion of the task compared to age-matched controls (Van den Broeck et al., 2021), suggesting that Aβ accumulation may more strongly affect the acquisition of new behavior rather than performance of established behaviors (Montgomery et al., 2011). Consistent with this suggestion, Soto et al. (2023) found that after the training phase, continuous versus discontinuous operant testing in these APP/PS1 mice had no effect on whether genotypes differed in working memory and attention, even though transgenic mice developed extensive Aβ loads.

Differences in cognitive performance of trained versus untrained mice may be due to a change in the brain networks that underlie performance following extended versus limited training histories. Repeated training on goal-oriented tasks for long periods of time can cause a shift in the neural network that maintains that task performance (Corbit and Balleine, 2000; Killcross and Coutureau, 2003). The goal-directed system relies heavily on hippocampal functioning, while stimulus-response tasks rely on the striatum (Goldfarb and Phelps, 2017). The early period of training and learning would be controlled by the hippocampal network, while the later stage, the habitual response, would be maintained by the striatal system. Aβ accumulation occurs in both networks, but the hippocampus is affected in early stages of AD, while striatal accumulation occurs only in late stages of AD (Hampel et al., 2021). Additionally, if this shift in neural networks occurs, there is some evidence that striatum is less affected by elevated glucocorticoid levels (Schwabe et al., 2010). Our mice showed extensive Aβ loads in the hippocampus and cortex (Soto et al., 2023), but we do not know if glucocorticoid action in these neural networks was altered.

The current results suggest that acute, pre-session stressor exposure does not reliably impair accuracy of DMTP and 3CSRT performance in APP/PS1 mice and suggests that the failure to obtain declines in performance accuracy over time (cf. Soto et al., 2023), is not simply due to a difference in stress exposure during tasks. It remains possible that conflicting results in cognitive impairment in APP/PS1 mice arise from a complex interaction of stress effects and training parameters. Future research might evaluate the effects of concurrent stressor exposure (e.g., using performance maintained by escape from aversive stimuli or exposing animals to a stressor during an operant task) and the possible differential sensitivity of learning versus performance (e.g., using a repeated acquisition task; Thompson and Moerschbaecher, 1978) in the development of deficits in APP/PS1 mice.

## Data availability statement

The raw data supporting the conclusions of this article will be made available by the authors, without undue reservation.

## Ethics statement

This animal study was reviewed and approved by the Institutional Animal Care and Use Committee at Texas Tech University.

## Author contributions

GD contributed to the conceptualization, maintained the daily testing and feeding, wrote the original manuscript, and conducted the stressor trials. BH and PS designed the experiments and supervised the work and managed the project. GD, BH, and PS contributed to the methodology development and data curation. GD and PS developed the ethograms for analysis and prepared the figures. PS analyzed the data – statistics. All authors contributed to the funding acquisition, review, and editing and article and approved the submitted version.

## Footnotes

1
http://predatorpee.com/
