# Supplementary material for: Acute stressors do not impair short-term memory or attention in an aged mouse model of amyloidosis
Source: Front Behav Neurosci. 2023 May 12;17:1151833. doi: 10.3389/fnbeh.2023.1151833 (PMC10213425; doi:10.3389/fnbeh.2023.1151833)

**Supplementary Materials**

Supplementary Figure 1. Overall Testing Timeline and Task Procedure Outlines. (A) Mice were weaned and genotyped around 3 weeks of age. Mice were assigned to either DMTP or 3CSRT task groups and trained and tested from weeks 16 to 72. During the final two weeks of testing, mice were tested normally for two days, received a stressor event immediately prior to testing on the third day, and resumed normal testing on the last two days. (B) DMTP procedural outline: Trials began with illumination of the house light followed by extension of one lever. If no response was made within 20-sec, the trial ended and a 10-sec intertrial interval (ITI) began. If the mouse pressed the lever, it retracted, and a delay of 0.1, 1, 2, 4, 8, 16, or 24 seconds began. Following the delay period, a nose-poke entry triggered the extension of the two levers. If no nose-poke was made within 20 s or a lever was not pressed within 20 s, the trial ended, and a 10-sec ITI began. If the mouse pressed the incorrect lever, the trial ended, and a 20-s timeout (TO) occurred during which all lights were off, and levers retracted. If the mouse pressed the correct lever, a 20-mg food pellet was dispensed followed by a 10-sec ITI. (C) 3CSRT task procedural outline: Each trial began with illumination of the house light and a 5-sec pre-stimulus period. One of the three nose-poke holes was then illuminated for a maximum of 1 s, and a nose-poke response during the illumination or within 5-sec after illumination produced delivery of a food pellet followed a 5-s ITI. Responses prior to illumination of the nose-poke light (premature responses), responses to a non-illuminated hole (incorrect responses), or failures to respond within 5-sec after light presentation (omission responses) produced a 15-sec ITI followed by a new trial.


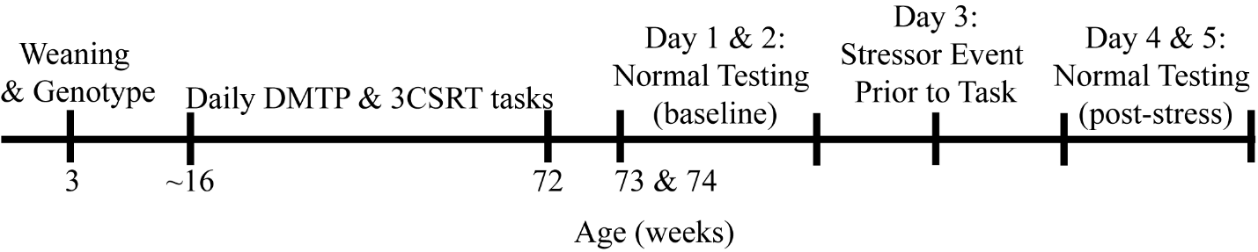


B

A


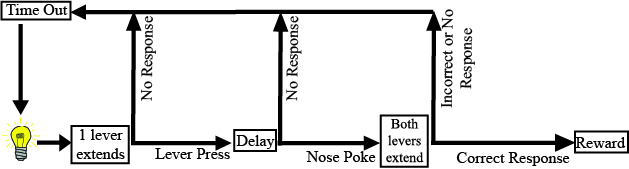


C


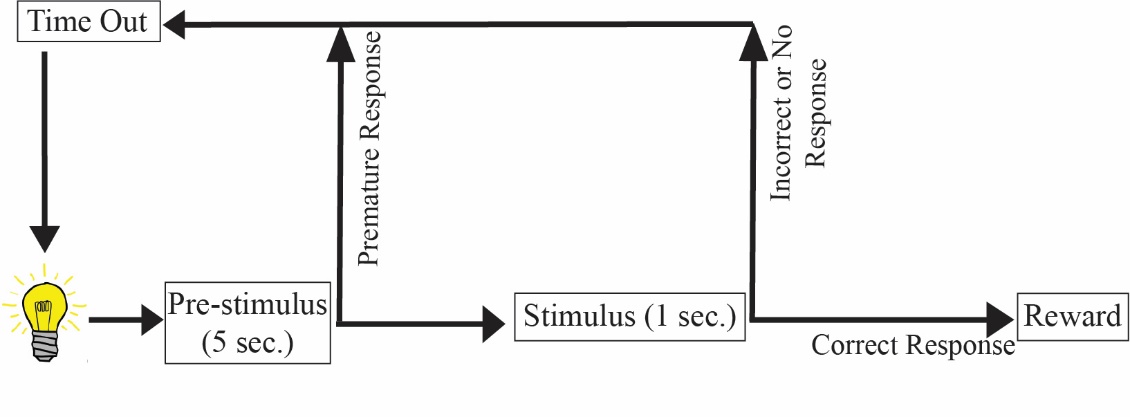


Supplementary Figure 2. Post-Stress Blood Corticosterone Levels. (A). Stressor by Genotype. Exposure to predator odor significantly increased plasma corticosterone F (1, 44) = 10.88, P = 0.0019, and transgenic mice had higher plasma corticosterone than non-transgenic F (1, 46) = 4.226, P = 0.045. There was no interaction between genotype and condition. Exposure to swimming significantly elevated plasma corticosterone F (1, 40) = 113.0, P < 0.001, but there was no impact of genotype nor was there an interaction. (B). Stressor by Genotype and Sex. Exposure to predator odor significantly increased plasma corticosterone F (1, 42) = 10.48, P = 0.0024, and transgenic mice had higher plasma corticosterone than non-transgenic F (1, 44) = 4.520, P = 0.0391. Sex did not impact results and there were no interactions. Exposure to swimming significantly elevated plasma corticosterone F (1, 38) = 118.6, P < 0.001, but there was no impact of genotype, sex, or any interactions.

A


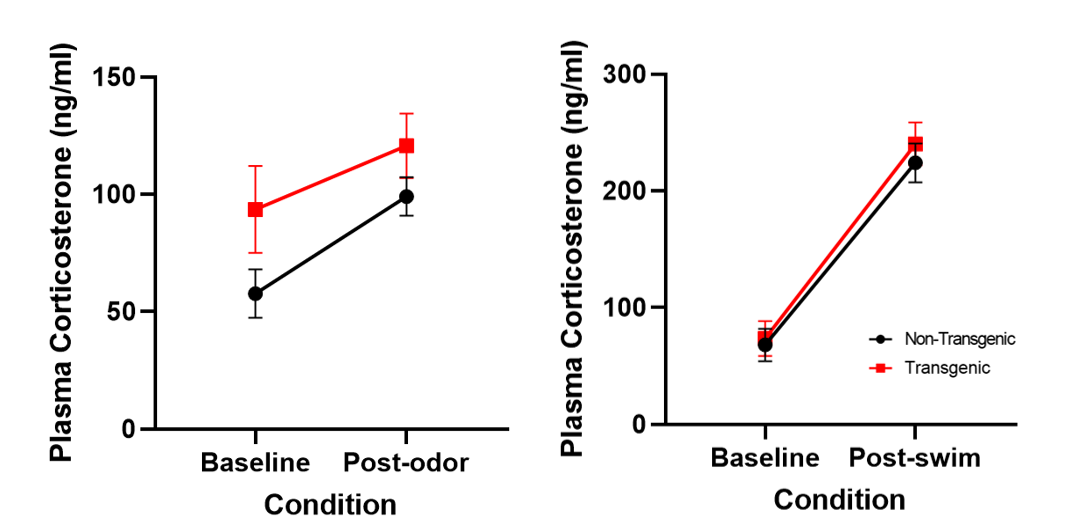


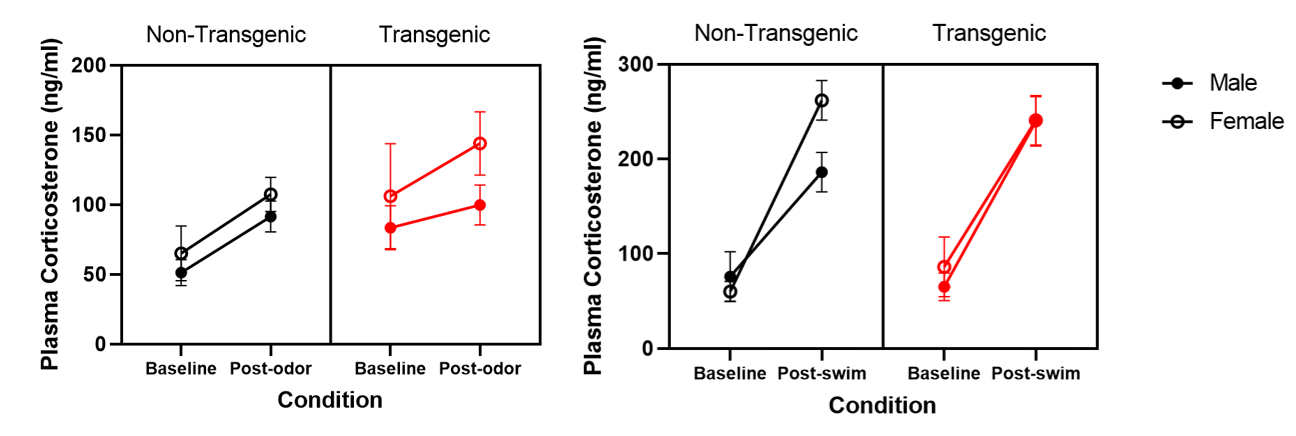


B

Supplementary Table 1. DMTP model including Sex as a predictor.

| **Predictor** | **Coefficient** | **SE** | **z** | **p** |
| --- | --- | --- | --- | --- |
| (Intercept) | 2.506 | 0.225 | 11.12 | < .001 |
| GenotypeTg | 0.153 | 0.335 | 0.46 | .647 |
| c.LogDelay | -2.363 | 0.228 | -10.38 | < .001 |
| SessionCategoryOn | 0.355 | 0.177 | 2.01 | .045 |
| SessionCategoryAfter | 0.050 | 0.131 | 0.38 | .703 |
| SexM | 0.582 | 0.322 | 1.81 | .071 |
| GenotypeTg:c.LogDelay | 0.003 | 0.340 | 0.01 | .992 |
| GenotypeTg:SessionCategoryOn | -0.271 | 0.255 | -1.06 | .287 |
| GenotypeTg:SessionCategoryAfter | -0.038 | 0.195 | -0.20 | .845 |
| c.LogDelay:SessionCategoryOn | -0.278 | 0.266 | -1.04 | .297 |
| c.LogDelay:SessionCategoryAfter | 0.332 | 0.200 | 1.66 | .096 |
| GenotypeTg:SexM | -0.844 | 0.468 | -1.80 | .071 |
| c.LogDelay:SexM | -0.036 | 0.327 | -0.11 | .913 |
| SessionCategoryOn:SexM | -0.146 | 0.259 | -0.56 | .575 |
| SessionCategoryAfter:SexM | 0.061 | 0.200 | 0.31 | .760 |
| GenotypeTg:c.LogDelay:SessionCategoryOn | 0.326 | 0.384 | 0.85 | .396 |
| GenotypeTg:c.LogDelay:SessionCategoryAfter | -0.107 | 0.297 | -0.36 | .719 |
| GenotypeTg:c.LogDelay:SexM | 0.442 | 0.473 | 0.93 | .351 |
| GenotypeTg:SessionCategoryOn:SexM | 0.548 | 0.365 | 1.50 | .134 |
| GenotypeTg:SessionCategoryAfter:SexM | 0.404 | 0.287 | 1.41 | .159 |
| c.LogDelay:SessionCategoryOn:SexM | 0.076 | 0.385 | 0.20 | .844 |
| c.LogDelay:SessionCategoryAfter:SexM | -0.326 | 0.299 | -1.09 | .275 |
| GenotypeTg:c.LogDelay:SessionCategoryOn:SexM | -0.519 | 0.546 | -0.95 | .342 |
| GenotypeTg:c.LogDelay:SessionCategoryAfter:SexM | -0.262 | 0.432 | -0.61 | .544 |

Supplementary Figure 3. Individual DMTP Proportion Correct Responses


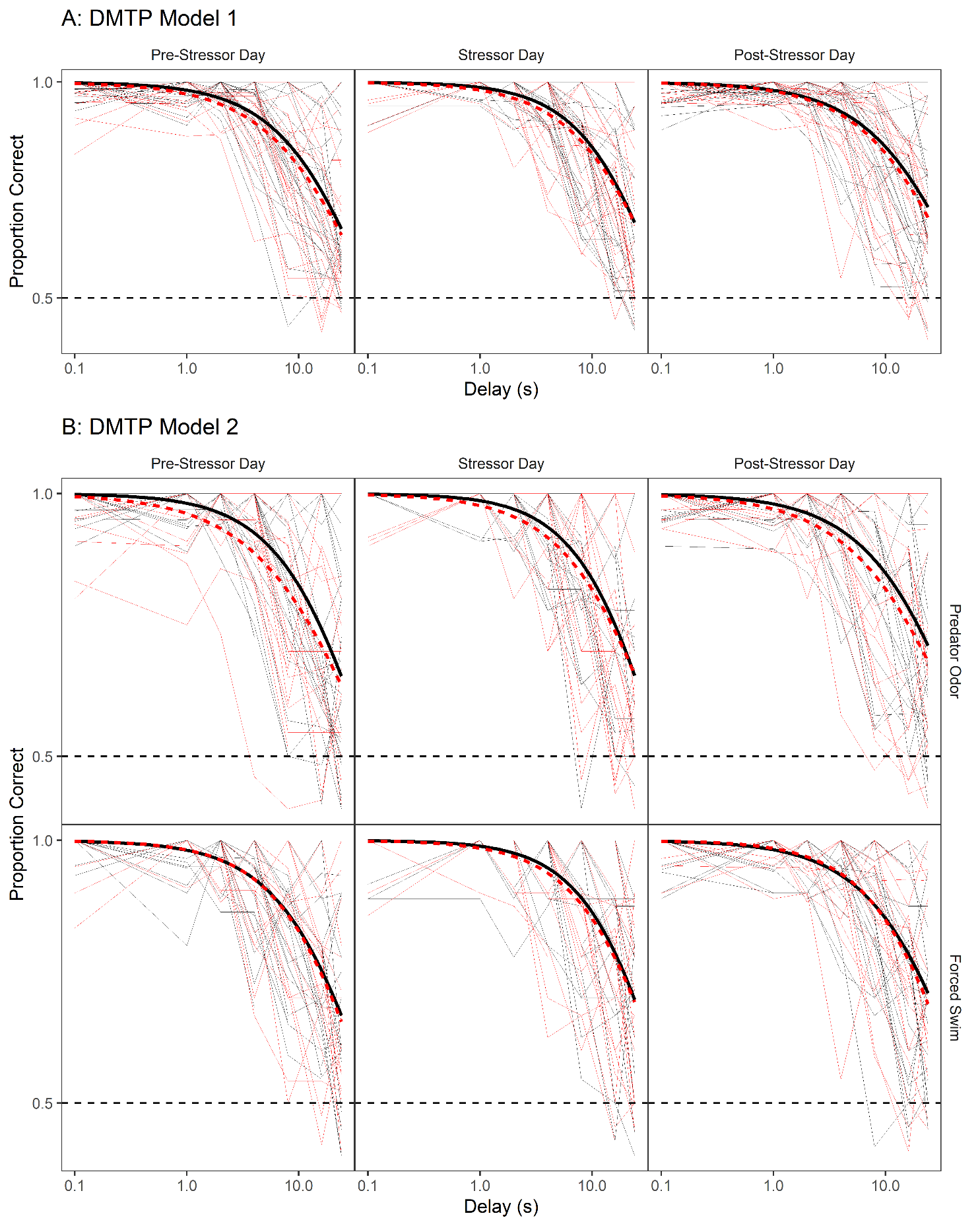


Supplementary Table 2. 3CSRTT model including Sex as a predictor.

| **Predictor** | **Coefficient** | **SE** | **z** | **p** |
| --- | --- | --- | --- | --- |
| (Intercept) | 3.056 | 0.221 | 13.82 | < .001 |
| GenotypeTg | 0.193 | 0.347 | 0.56 | .578 |
| SessionCategoryOn | 0.200 | 0.146 | 1.37 | .170 |
| SessionCategoryAfter | 0.078 | 0.116 | 0.67 | .502 |
| SexM | 0.556 | 0.326 | 1.70 | .089 |
| GenotypeTg:SessionCategoryOn | -0.048 | 0.228 | -0.21 | .833 |
| GenotypeTg:SessionCategoryAfter | 0.051 | 0.179 | 0.28 | .777 |
| GenotypeTg:SexM | -0.025 | 0.503 | -0.05 | .961 |
| SessionCategoryOn:SexM | -0.269 | 0.251 | -1.07 | .284 |
| SessionCategoryAfter:SexM | -0.285 | 0.209 | -1.36 | .173 |
| GenotypeTg:SessionCategoryOn:SexM | 0.123 | 0.374 | 0.33 | .742 |
| GenotypeTg:SessionCategoryAfter:SexM | 0.232 | 0.310 | 0.75 | .453 |

Supplementary Figure 4. Individual 3CSRTT Proportion Correct Responses


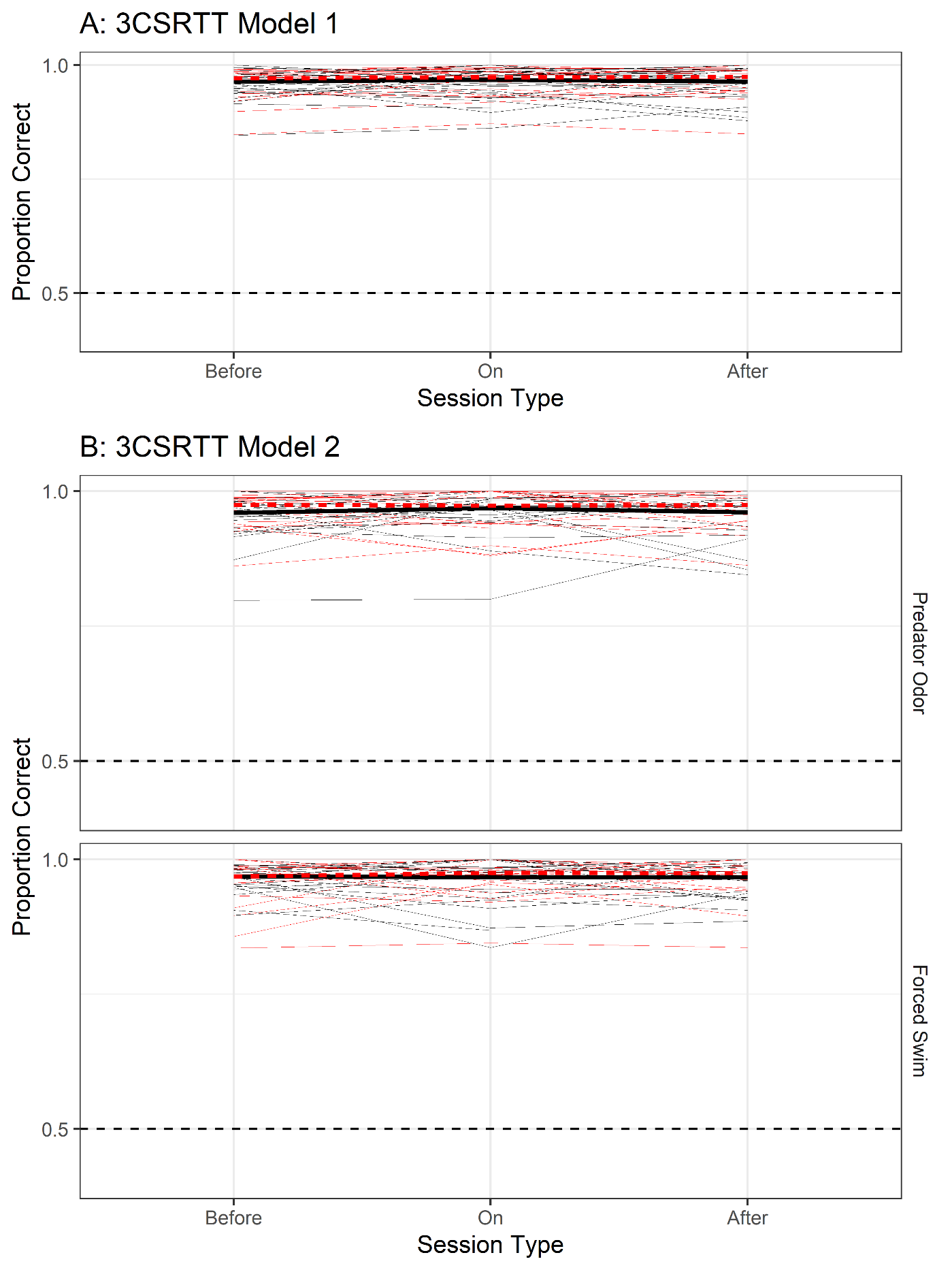

Supplement: Supplementary file 1 [file Data_Sheet_1.docx]
